# Visualization of Sensory Neurons and Their Projections in an Upper Motor Neuron Reporter Line

**DOI:** 10.1371/journal.pone.0132815

**Published:** 2015-07-29

**Authors:** Barış Genç, Amiko Krisa Bunag Lagrimas, Pınar Kuru, Robert Hess, Michael William Tu, Daniela Maria Menichella, Richard J. Miller, Amy S. Paller, P. Hande Özdinler

**Affiliations:** 1 Davee Department of Neurology and Clinical Neurological Sciences, Northwestern University, Feinberg School of Medicine, Chicago, IL, United States of America; 2 Department of Molecular Pharmacology and Biological Chemistry, Northwestern University, Feinberg School of Medicine, Chicago, IL, United States of America; 3 Robert H. Lurie Comprehensive Cancer Center, Northwestern University, Feinberg School of Medicine, Chicago, IL, United States of America; 4 Departments of Dermatology and Pediatrics, Northwestern University, Feinberg School of Medicine, Chicago, IL, United States of America; 5 Skin Disease Research Center, Northwestern University, Feinberg School of Medicine, Chicago, IL, United States of America; 6 Center for Genetic Medicine, Northwestern University, Feinberg School of Medicine, Chicago, IL, United States of America; 7 Cognitive Neurology and Alzheimer's Disease Center, Northwestern University, Chicago, IL, United States of America; National Institute of Health, UNITED STATES

## Abstract

Visualization of peripheral nervous system axons and cell bodies is important to understand their development, target recognition, and integration into complex circuitries. Numerous studies have used protein gene product (PGP) 9.5 [a.k.a. ubiquitin carboxy-terminal hydrolase L1 (UCHL1)] expression as a marker to label sensory neurons and their axons. Enhanced green fluorescent protein (eGFP) expression, under the control of *UCHL1* promoter, is stable and long lasting in the UCHL1-eGFP reporter line. In addition to the genetic labeling of corticospinal motor neurons in the motor cortex and degeneration-resistant spinal motor neurons in the spinal cord, here we report that neurons of the peripheral nervous system are also fluorescently labeled in the UCHL1-eGFP reporter line. eGFP expression is turned on at embryonic ages and lasts through adulthood, allowing detailed studies of cell bodies, axons and target innervation patterns of all sensory neurons *in vivo*. In addition, visualization of both the sensory and the motor neurons in the same animal offers many advantages. In this report, we used UCHL1-eGFP reporter line in two different disease paradigms: diabetes and motor neuron disease. eGFP expression in sensory axons helped determine changes in epidermal nerve fiber density in a high-fat diet induced diabetes model. Our findings corroborate previous studies, and suggest that more than five months is required for significant skin denervation. Crossing UCHL1-eGFP with hSOD1^G93A^ mice generated hSOD1^G93A^-UeGFP reporter line of amyotrophic lateral sclerosis, and revealed sensory nervous system defects, especially towards disease end-stage. Our studies not only emphasize the complexity of the disease in ALS, but also reveal that UCHL1-eGFP reporter line would be a valuable tool to visualize and study various aspects of sensory nervous system development and degeneration in the context of numerous diseases.

## Introduction

The cellular and molecular basis of peripheral nervous system (PNS) development, target recognition, innervation, and establishment of circuitries and feedback loops have been an area of active research [1,2]. The interaction between the PNS and the central nervous system (CNS) ensures proper function of complex circuitries. It is not easy to visualize and trace the neurons and axons of the PNS *in vivo*, and it is especially challenging to monitor them in live model systems. However, our ability to monitor the axons and cells of the PNS has the potential to reveal important information during development and adulthood, and within the context of disease.

Ubiquitin carboxy-terminal hydrolase L1 (UCHL1), formerly known as protein gene product (PGP) 9.5 [3–5], is used as a marker of PNS axons. UCHL1 is highly expressed in neurons of the sensory ganglia [6–9], sympathetic ganglia [7,9], enteric nervous system (ENS) [9–12], retina [9,13], as well as spermatogonia and sertoli cells in testis [14]. In trigeminal ganglia (TG) and dorsal root ganglia (DRG) neurons, UCHL1 protein can be detected as early as embryonic day (E) 10.5 in the developing mouse [8]. In addition to cell bodies, UCHL1 can be detected in neuronal projections, allowing investigation of developing limb bud [15] and cutaneous innervation [16,17]. The strong and persistent UCHL1/PGP9.5 expression in the axons and cell bodies of peripheral neurons made it an ideal candidate as a marker to visualize and study the timing and extent of axonal projections in the periphery, and visceral organs.

We recently generated the UCHL1-eGFP reporter line by using the *UCHL1* promoter to drive enhanced green fluorescent protein (eGFP) expression in the mouse [18], and previously reported that eGFP expression labels corticospinal motor neurons (CSMN) in layer V of the motor cortex. In the spinal cord, all spinal motor neurons (SMN) are eGFP-positive (eGFP^+^) at birth, however in the adult, eGFP expression becomes restricted to a subset of small diameter alpha and gamma motor neurons that are resistant to degeneration in amyotrophic lateral sclerosis (ALS) [18]. Since UCHL1 expression is mostly studied in the periphery and UCHL1 expression is used as a marker to label sensory axons both during development and adulthood, here we characterize the eGFP expression in the PNS of UCHL1-eGFP reporter line. We find that eGFP expression in the periphery strongly correlates with the UCHL1 expression *in vivo*, and thus can replace the need to section and immunostain sections with UCHL1 antibody to visualize axon fibers and cell bodies in the periphery. eGFP expression is observed during embryonic ages and is persistent through adulthood without a change in intensity and pattern. Since eGFP expression is observed in the axons, details of organ skin innervation can be monitored in live animals.

Movement is a complicated task, as it requires proper input both from the motor neurons in the cortex and spinal cord, but also the peripheral neurons, which carry information from the environment to the CNS and precisely modulate motor function. UCHL1-eGFP reporter line is unique because it genetically labels motor neurons in the CNS as well as the sensory neurons in the PNS, allowing visualization and detailed investigation of these two different neuron pools in the same model system. Such reporter lines are required to study complex circuitries both at a cellular and molecular level. Using UCHL1-eGFP mice, we generated a reporter line of ALS disease to investigate the potential involvement of sensory nervous system defects, and our findings not only further emphasize ALS as a multi-systems disorder, but also report progressive degeneration of peripheral neurons, albeit at later time points.

## Materials and Methods

### Mice

This study was carried out in strict accordance with the recommendations in the Guide for the Care and Use of Laboratory Animals of the National Institutes of Health. All animal procedures were approved by the Northwestern University Animal Care and Use committee and comply with the standards of the National Institutes of Health (Protocol Numbers: 2011–1446, 2011–2657). In this study, wild type (WT) and hSOD1^G93A^ mice (The Jackson Laboratory), and UCHL1-eGFP reporter mice (generated by the Özdinler Lab at the Northwestern Targeted Mutagenesis Core facility) of either sex were used. All mice were on C57BL/6 background.

### Generation of UCHL1-eGFP reporter mice

UCHL1-eGFP reporter mice were generated as previously described [18] (now at the Jackson Laboratory: strain name C57BL/6-Tg(Uchl1-EGFP)G1Phoz/J). Newborn pups were initially screened for eGFP expression in the spinal cord and prefrontal cortex (Blue- Star GFP, NightSea) at postnatal day (P) 0–P3. Tail DNA was analyzed to detect eGFP by PCR using forward 5’-CCTACGGCGTGCAGTGCTTCAGC-3’ and reverse 5’-CGGCGAGCTGCACGCTGCCGTCCTC-3’ primers. G1, and G7 lines showed strong eGFP expression that was evident with consistent pattern and intensity of expression from litter to litter for >8 generations and were used for further analysis in this study.

### Generation of hSOD1^G93A^-UeGFP mice

Transgenic hemizygous males expressing a high copy number of the human SOD1 gene with the G93A mutation [B6SJL-Tg(SOD1*G93A)1Gur/J; The Jackson Laboratory] were crossed with hemizygous UCHL1-eGFP females (lines G1 and G7) to generate hSOD1^G93A^-UeGFP and WT-UeGFP (control) mice. Transgenic mice were identified by PCR amplification of DNA extracted from tail as previously described [18]. Mice at P30 (presymptomatic), P60 (early symptomatic), P90 (symptomatic), and P120 (end-stage) were used for analysis.

### Diabetes induction and Glucose Tolerance Test

Mice were fed a high fat diet (HFD) consisting of 60% fat, 20% carbohydrate, and 20% protein (total 5.2 kcal/g, D12492 60% kcal diet, Research Diets Inc, NJ) as described previously [19]. Mice were placed on the HFD diet at 6 weeks, and kept on the diet for 20 weeks with serial weights and the presence of insulin resistance monitored by metabolic analyses.

At 22 weeks, mice were fasted overnight (16 h) with free access to water before injecting intraperitoneally with D-glucose (2 g/kg). Blood was obtained by tail nick at baseline, 30, 60, and 120 min after glucose administration [19]. The animal was briefly anesthetized by isoflurane for 3–5 min, whole blood obtained from the intraorbital retrobulbar plexus was allowed to clot at RT for 30 min before centrifugation at 4°C to separate serum from clotted blood component, and serum was stored at -80°C until insulin was measured using Ultra Sensitive Mouse Insulin ELISA Kit (Crystal Chem Inc., Downers Grove, IL). Absorbance was read at 450 nm in an EL808 microplate reader (BIO-TEK Inc., Winooski, VT) linked with the KC Junior program.

### Whole mouse imaging

Whole mice were deeply anesthetized using ketamine (90 mg/kg) with xylazine (10 mg/kg), and transcardially perfused with 4% PFA in PBS. Fur was removed from the back and hind legs using commercial hair removal lotion. Mice were observed under a Nikon SMZ 1500 dissecting microscope attached to an Intensilight C-HGFI light source with a GFP-B filter cube.

### Histology

Neonatal and adult mice were deeply anesthetized using ketamine (90 mg/kg) with xylazine (10 mg/kg), and transcardially perfused with 4% PFA in PBS. Embryos were collected from dams under anaesthesia using a Caesarean section. The sensory ganglia, autonomic ganglia, intestines, gonads, whisker pads, footpads and hairy skin were removed intact and postfixed (4% PFA, overnight) and stored in PBS with sodium azide (0.01%) at 4°C. 50 μm-thick free-floating sections were cut using a vibratome (Leica) or a sliding microtome and collected in 12-well plates. Alternatively, tissues were cryoprotected in 30% sucrose, embedded in OCT (Sakura Finetek), and 10–20 μm-thick sections were cut in a CM 3050S cryostat (Leica), and serial sections were immediately mounted on slides. Sections were either used directly for imaging or were processed for immunocytochemical analysis.

### Clearing eGFP embryos

eGFP^+^ embryos were cleared in SeeDB as previously described [20]. Briefly, embryos were fixed in 4% PFA in PBS and embedded in 1% agarose. SeeDB was prepared by dissolving D(-)-fructose in H_2_O at 65°C and adding α-thioglycerol after cooling to room temperature, to achieve a final concentration of 80.2% (wt/wt) fructose and 0.5% α-thioglycerol. Embryos were incubated in increasing concentrations of 20%, 40% and 60% fructose for 4–8 hrs each with gentle shaking, until they were finally transferred into the SeeDB for at least 24 hours before imaging.

### Immunocytochemistry and cellular staining

Primary antibodies used are as follows: chicken polyclonal anti-GFP (1:1000, Abcam, MA), rabbit polyclonal anti-GFP (1:1000, Invitrogen, NY), rabbit polyclonal anti-UCHL1 (1:1000, Proteintech, IL), chicken polyclonal anti-neurofilament-heavy (NF-H, 1:1000, Millipore, MA), rabbit polyclonal anti-parvalbumin (PV, 1:5000, Swant, Switzerland), rabbit polyclonal anti-calcitonin gene-related peptide (CGRP, 1:1000, Chemicon, MA). Far red-conjugated isolectin B_4_ (IB_4_, 1:500, Invitrogen, NY) was included with anti-GFP primary antibody. Appropriate secondary fluorescent antibodies (1:1000, AlexaFluor-488-conjugated, Invitrogen, NY; FITC-conjugated, Invitrogen, NY; or Cy3-conjugated, Millipore, MA) were added to blocking solution at room temperature for 2 h in the dark. All antibodies used were characterized previously, and specificity validated by using controls processed alongside other samples omitting the primary antibodies. GFP immunocytochemistry was further validated by using WT mouse tissue that does not contain the eGFP transgene.

### Wholemount immunohistochemistry of hairy skin

Wholemount immunohistochemistry of hairy skin was performed as previously described [21]. Briefly, back hairy skin from adult mice was treated with commercial hair removal lotion and stripped with laboratory tape. Skin was fixed flat in 4% PFA in PBS and cut into small pieces. Skin blocks were then washed in 0.3% PBST every 30 min for 5 hrs, and incubated with anti-GFP antibody in 0.3% PBST containing 5% serum and 20% DMSO for 2 days. Skin tissue was washed every 30 min for 8 hrs with 0.3% PBST and incubated in secondary antibody diluted in 0.3% PBST containing 5% serum and 20% DMSO for 2 days. After washing every 30 min for 8 hrs with 0.3% PBST, skin tissue was dehydrated in 50% methanol for 5 min and three times in 100% methanol for 20 min each. Finally wholemount skin was cleared in 1:2 solution of benzyl alcohol:benzyl benzoate (BABB) for 20 min, cover slipped and imaged.

### Data collection and imaging

Low-magnification images were acquired using a SMZ 1500 fluorescent dissecting scope (Nikon) for qualitative analysis of GFP expression pattern at different ages. An Eclipse TE2000-E epifluorescent microscope (Nikon) and a TCS SP5 confocal microscope (Leica) were used for high magnification images. Z-stacks were processed using ImageJ (National Institutes of Health) to generate maximum intensity projections.

### Quantification

Lumbar (L) 3 & L4 DRGs were isolated from P30 UCHL1-eGFP reporter mice (*n* = 3) to quantify eGFP co-localization with molecular markers UCHL1, PV and IB_4_ using 10 μm thick frozen sections. Neurons were imaged and counted. Results were represented as average percent co-localization ± SEM.

Footpads collected from UCHL1-eGFP mice on HFD and RD, and hSOD1^G93A^-UeGFP, and littermate control mice (WT-UeGFP) were used for the quantification of epidermal innervation density. 20 μm thick frozen sections were used from HFD (*n* = 6) and RD (*n* = 5), or hSOD1^G93A^-UeGFP and WT-UeGFP mice at P30 (*n* = 6), P60 (*n* = 3), P90 (*n* = 3), and P120 (*n* = 6). eGFP^+^ axons in the epidermis were imaged and counted, and length of the epidermis was measured using NIS-Elements software (Nikon). Results were represented as average number of axons per mm length of epidermis ± SEM.

### Statistical analysis

Statistical data analyses were performed using Prism software (GraphPad Software). D’Agostino and Pearson normality test was performed on all datasets. Statistical differences between two groups were determined using Student’s *t* test. Statistically significant differences were taken at *P* < 0.05.

## Results

### UCHL1-eGFP reporter mice recapitulates endogenous UCHL1 expression pattern in the peripheral nervous system

**Fig 1:**
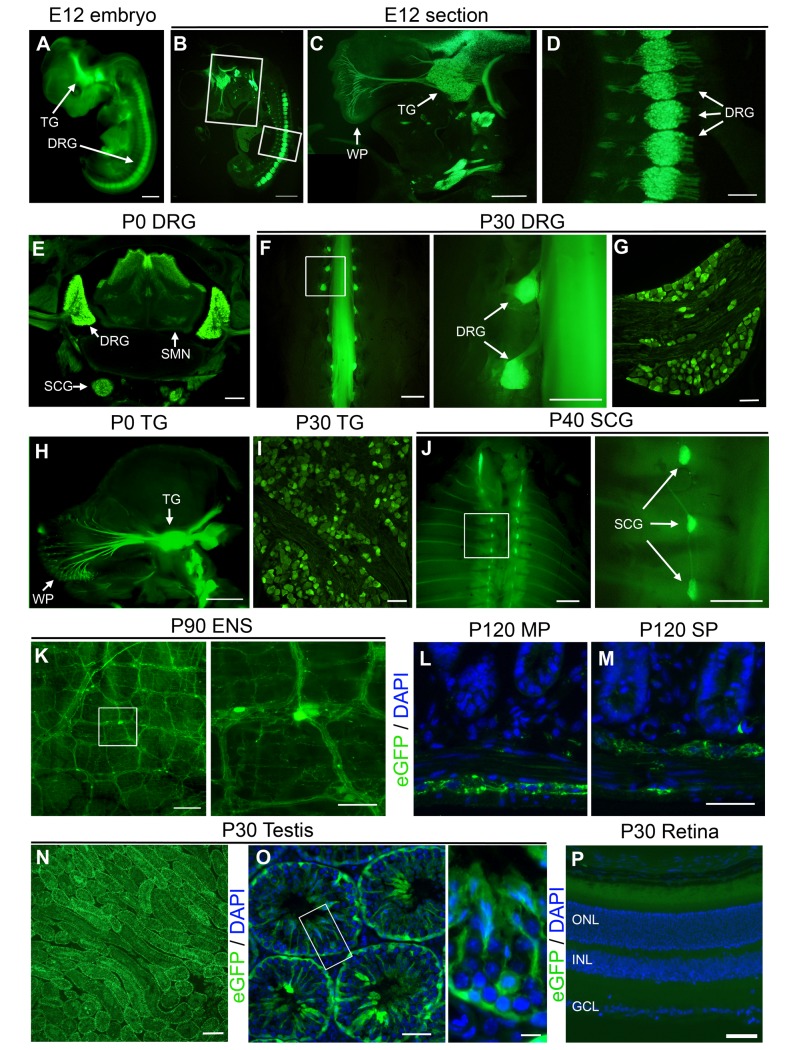
UCHL1-eGFP reporter line allows visualization of peripheral nervous system *in vivo*. (A) Enhanced green fluorescent protein (eGFP) expression is evident at embryonic day (E) 12 in UCHL1-eGFP mice. (B-D) Cross-section through the E12 embryo showing eGFP^+^ trigeminal ganglia (TG; C) and dorsal root ganglia (DRG; D) neurons. (E) Cross-section of the postnatal day (P) 0 trunk reveals eGFP^+^ DRG neurons, their axons, sympathetic chain ganglia (SCG), and spinal motor neurons (SMN) in the ventral horn of the spinal cord. (F) eGFP expression in the adult DRG. Insert enlarged to the right. (G) Cross section of DRG section reveals eGFP+ neurons. (H) Sagittal-section of the P0 head showing eGFP^+^ TG neurons and their axons projecting to the whisker pad and brainstem. (I) Cross section of adult TG. (J) eGFP^+^ SCG in the exposed adult thoracic cavity. Insert enlarged to the right. (K) Open-book prep of wholemount intestines displays a network of eGFP^+^ enteric nervous system (ENS) neurons and their axons. Insert enlarged to the right. (L-M) Cross-sections of intestines show ENS neurons in both the myenteric plexus (MP; L) and submucosal plexus (SP; marked with eGFP expression; M). (N-O) eGFP is expressed in the testis. (P) eGFP is not expressed in the retina. WP: whisker pad, ONL: outer nuclear layer, INL: inner nuclear layer, GCL: ganglion cell layer. Scale bars A, B, F inset and J inset 1 mm; C 500 μm; D, E, K, N 200 μm; F, H, J 2 mm; G, I, K inset, L, M, O 50 μm; O inset 10 μm; and P 100 μm.

To genetically label distinct populations of neurons and allow their immediate visualization *in vivo*, we recently generated UCHL1-eGFP reporter line, which expresses eGFP under the control of *UCHL1* promoter. We previously showed that in the CNS, eGFP expression is restricted to CSMN in the motor cortex and degeneration-resistant SMN in the spinal cord [18]. However, since high levels of UCHL1 expression in the sensory nervous system is used as a marker to trace the sensory neurons in the periphery [8,9], we next investigated whether eGFP expression is present in the PNS in the UCHL1-eGFP mice (Fig 1).

Developing DRG and TG are eGFP^+^ at E12 (Fig 1A–1D). Tissue clearing enables visualization of axon fibers in an intact embryo (Fig 1A): TG neurons innervating the whisker pad (WP; Fig 1C), and the DRG axons (Fig 1D) become visible. Cross sections through the trunk of a newborn pup show spinal cord, DRG, and sympathetic chain ganglia (SCG) in one plane, and reveal that in addition to the SMN located in the ventral horn of the spinal cord [18], the DRG and SCG cell bodies are also eGFP^+^ (Fig 1E). Stability of eGFP expression is visible both in intact (Fig 1F), and in cross sections of DRG (Fig 1G) in the adult mice. Similarly, TG neurons are also eGFP^+^ at birth (Fig 1H) and in adulthood (Fig 1I).

UCHL1 expression is previously reported in SCG which is part of the autonomic nervous system, and the ENS [7,9–12]. We find that eGFP expression in the SCG is strong enough to be detected even in an intact adult mouse (Fig 1E and 1J). Interestingly, the details of the ENS and the innervation patterns of both circular and longitudinal muscles of the intestine become visible in the UCHL1-eGFP mice (Fig 1K). Myenteric plexus (MP; Fig 1L) and submucosal plexus (SP; Fig 1M) soma and axonal arbors are visualized without a need for immunocytochemical labeling.

In addition to the peripheral nervous system, testes are known to express UCHL1 [14], and were strongly labeled by eGFP in the UCHL1-eGFP mice (Fig 1N). High magnification images of sections through seminiferous tubules show that the eGFP is present in the spermatagonia and the supporting Sertoli cells, but not in Leydig cells (Fig 1O). Retina, however, does not display eGFP expression (Fig 1P), even though it is known to express UCHL1 [9,13].

### Nociceptive sensory neurons display bright eGFP expression

**Fig 2:**
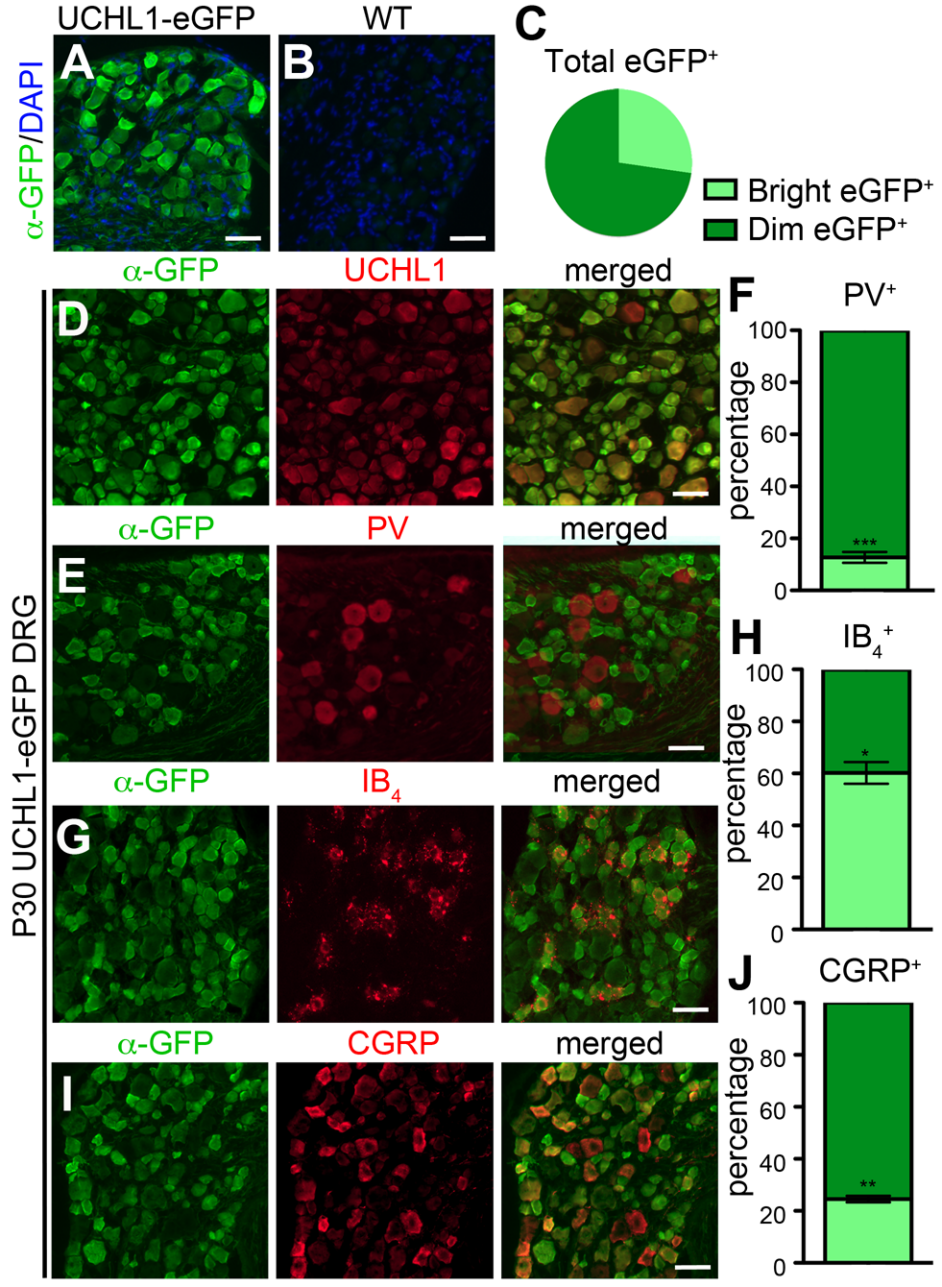
All DRG neurons are eGFP+ and nociceptive neurons display bright GFP. (A-B) eGFP is expressed in all DRG neurons in UCHL1-eGFP reporter mice (A) and is completely absent in the wild type (WT) mice (B). (C) Pie-chart graph showing distribution of eGFP^+^ neurons. (D) eGFP co-localizes with UCHL1 in P30 DRG neurons. (E) Co-localization of bright eGFP^+^ with Parvalbumin (PV), a marker for proprioceptive neurons. (F) Bar graph representation of average percentage of bright and dim eGFP^+^ DRG neurons among PV^+^ neurons. (G) Co-localization of bright eGFP^+^ with Isolectin IB_4_, a marker for nociceptive neurons. (H) Bar graph representation of average percentage of bright and dim eGFP^+^ DRG neurons among IB_4_^+^ neurons. (I) Co-localization of bright eGFP^+^ with calcitonin gene related peptide (CGRP), a marker for nociceptive neurons. (J) Bar graph representation of average percentage of bright and dim eGFP^+^ DRG neurons among CGRP^+^ neurons. Bar graphs represent mean ± SEM. Student’s *t*-test, * *P* < 0.05, ** *P* < 0.01, *** *P* < 0.001. Scale bars A-B,D,E,G,I 100 μm.

eGFP expression is present in all neurons (100%; *n* = 3924 neurons) in the P30 lumbar DRG of the UCHL1-eGFP reporter line (Fig 2A), and not detected in the control WT littermates (Fig 2B). Close inspection of the DRG reveals that sensory neurons vary both in size and also in the intensity of eGFP and UCHL1 expression. Even though UCHL1 is used as a marker for all sensory neurons, similar to eGFP expression it displayed varied levels of intensity. Quantitative analysis of eGFP expression showed that even though all DRG neurons express eGFP, about 1/4^th^ of them are relatively brighter (Fig 2C; 27 ± 1% bright, 73 ± 1% dim; *P* < 0.0001). To investigate the possibility that these very bright GFP+ neurons represent a specific subpopulation of sensory neurons, we performed co-labeling studies using molecular markers for different classes, such as isolectin B_4_ (IB_4_), calcitonin gene related peptide (CGRP), and parvalbumin (PV), which are markers for peptidergic [22,23] and non-peptidergic [24] nociceptive, and proprioceptive [25,26] neurons, respectively (Fig 2E and 2G, and 2I). 64 ± 3% of bright eGFP^+^ neurons expressed CGRP (*n* = 2397 neurons; *P* = 0.002202173), 44 ± 3% co-localized with IB_4_ (*n* = 1687 neurons), and only 3% were PV^+^ (*n* = 5279 neurons; *P* < 0.0001), suggesting that the very bright neurons were mainly nociceptive, but not propriceptive. However, all DRG neurons expressed eGFP, albeit at different levels. Out of all the PV^+^ cells 13% were bright and 87% of dim (Fig 2F; *n* = 1419 neurons; *P* < 0.0001), but there was not a single PV^+^ neurons that did not express eGFP. Likewise, out of all the IB_4_^+^ neurons, 60 ± 4% were bright (Fig 2H; *n* = 1188 neurons; *P* = 0.0246), and out of all CGRP^+^ neurons 25 ± 1% were bright (Fig 2J; *n* = 1539 neurons, *P* < 0.0001). These quantitative analyses revealed that all DRG neurons were genetically labeled with eGFP expression in the UCHL1-eGFP mice, and mainly the nociceptive neurons displayed very bright eGFP expression.

### Peripheral axons become visible with eGFP expression *in vivo*

**Fig 3:**
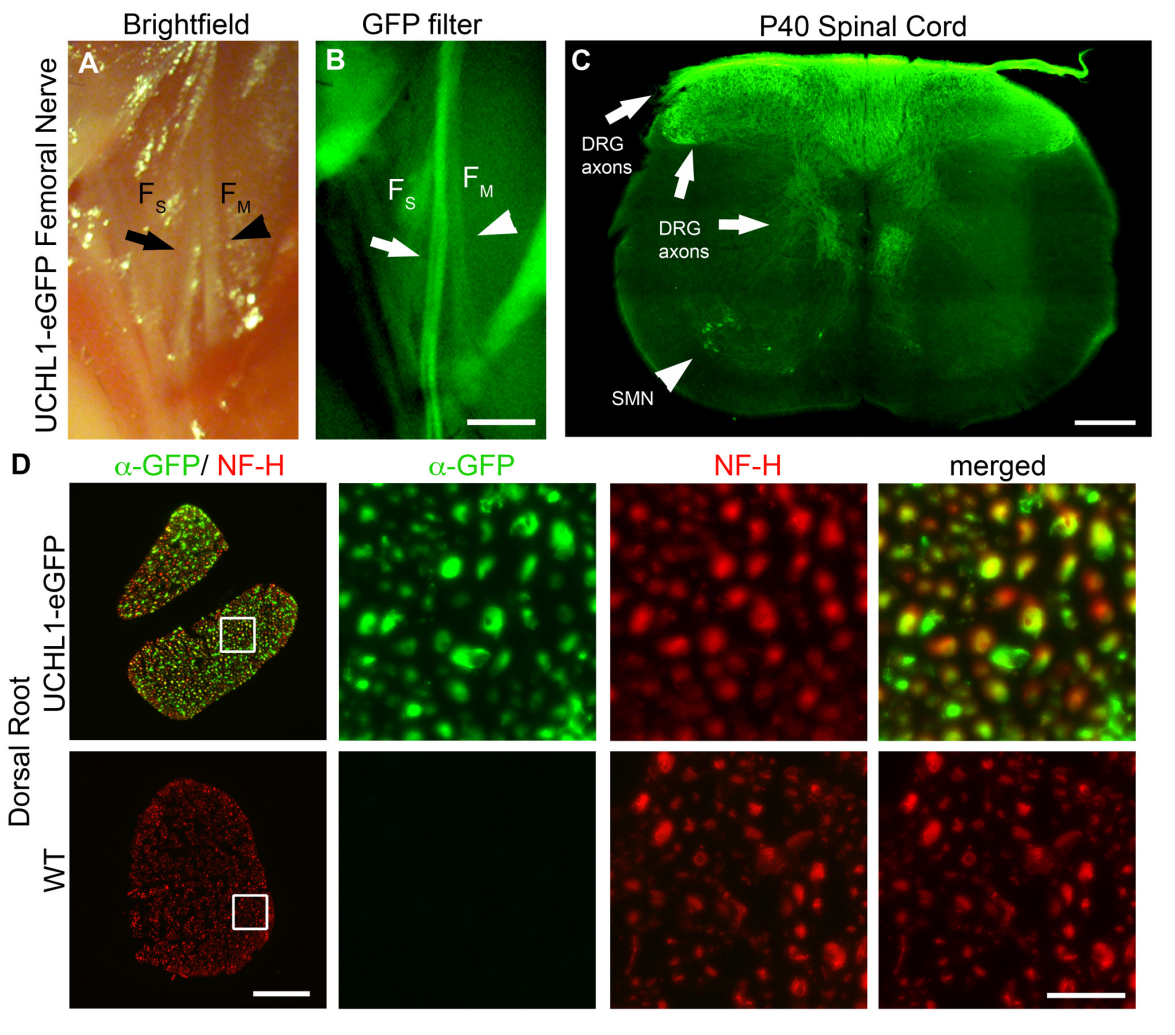
Sensory axons are eGFP^+^. (A-B) eGFP expression in the axons of sensory branch of the femoral nerve (F_S_, arrows) makes it visible *in vivo*. F_M_: femoral nerve motor branch (arrowheads). (C) DRG axons innervating the dorsal and ventral horn of the spinal cord are labeled by eGFP (arrows). Spinal motor neurons (SMN) in the ventral horn are also GFP^+^ (arrowhead). (D) Cross-sections of the dorsal root show complete overlap between neurofilament-H (NF-H) labeled axons and eGFP expression in the UCHL1-eGFP mice, and there is no GFP in the WT mice. Scale bars A,B 1 mm; C 250 μm; D 50 μm; D inset 20 μm.

We next investigated if eGFP was present in the axons, and whether *de novo* fluorescence would be strong enough to visualize axon tracts and individual fibers. We find that in the UCHL1-eGFP mice eGFP expression is present not only in the cell bodies (Fig 2A), but also in the peripheral (Fig 3A and 3B) and central (Fig 3C) projections of the DRG. When the femoral nerve is exposed, eGFP expression distinguishes the sensory branch (F_S_) from the motor branch (F_M_) in an intact mouse (Fig 3A and 3B). In addition, both the SMN located in the ventral horn of the spinal cord (arrowhead) and the central projections of DRG (arrows) can be visualized in the same section of the DRG (Fig 3C). However, since CSMN are also genetically labeled with eGFP, it is not possible to discern DRG axons and the branches of the corticospinal tract, innervating spinal targets. Cross-sections through the dorsal root reveal that all sensory axons labeled by neurofilament-H (NF-H) are also eGFP^+^ (Fig 3D), further demonstrating the presence of fluorescence in individual axon fibers.

**Fig 4:**
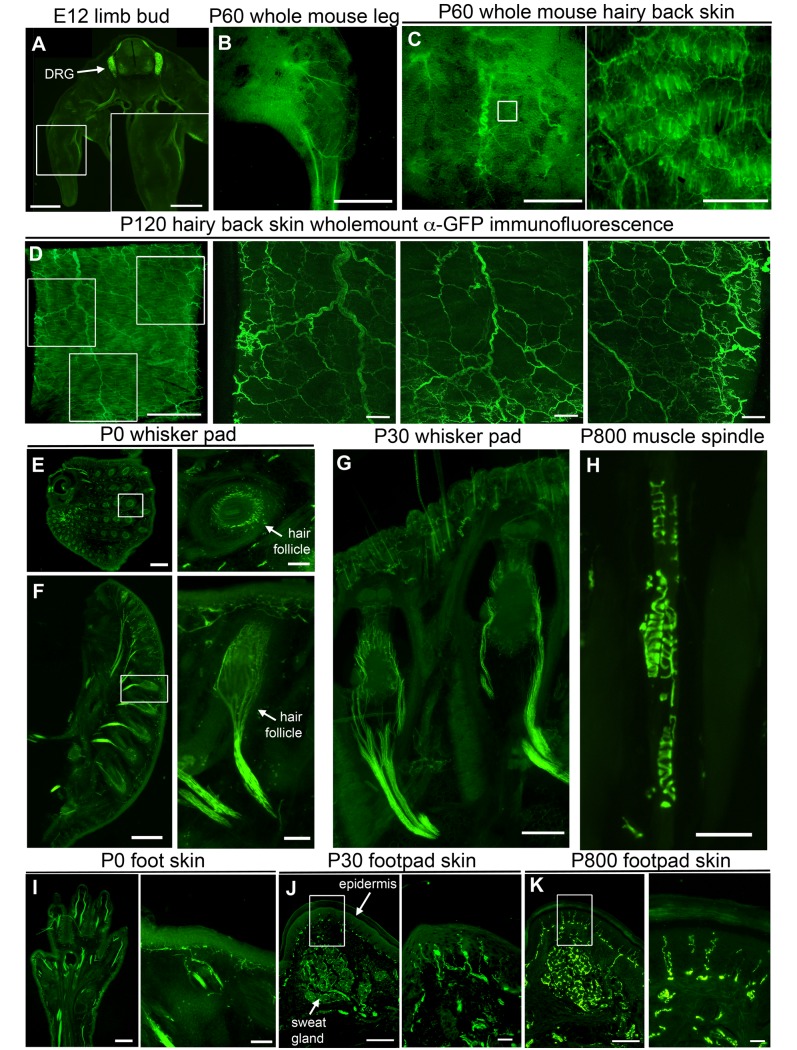
eGFP labels projections of sensory neurons in the periphery. (A-D) eGFP^+^ dorsal root ganglia (DRG) axons can be seen extending into the developing limb bud (A), in the depilated leg of the whole adult mouse (B), in the hairy back skin of the whole adult mouse both in section (C) and wholemount (D). Insert enlarged to the right. (E-G) UCHL1-eGFP reporter mouse can be used to study innervation of hair follicles in whisker pads of P0 (E-F) or adult mice (G). Insert enlarged to the right. (H) Proprioceptive nerve endings in muscle spindles are eGFP^+^. (I-K) eGFP^+^ axons can be used to study innervation of the P0 foot (I), or adult footpad epidermis and sweat glands (J, K). Insert enlarged to the right. Scale bars A, D 1 mm; A inset, B, C, E, F, G, I 500 μm; C inset, E inset, F inset, I inset 100 μm; D inset, J, K 200 μm; H 50 μm; J inset, K inset 25 μm.

Peripheral axons extending towards their targets in the developing limbs are eGFP^+^ during embryonic development (Fig 4A). When a depilated whole adult mouse is observed under a fluorescent dissecting microscope, deep nerve bundles in the leg (Fig 4B) and hairy back skin (Fig 4C) can be seen branching and extending towards their peripheral targets even at low magnification. Even though details of fine nerve endings on skin surface become apparent at high magnification (Fig 4C inset), wholemount immunocytochemistry using anti-GFP antibody (α-GFP) reveals very precise details of axon bundles entering the skin and splitting into finer branches (Fig 4D).

Whisker pad innervation offers a good tool to study axon-target interactions during development [27,28]. TG axons innervating the whisker pad are eGFP^+^ as early as E12 (Fig 1C), and individual axons surrounding hair follicles are observed in great detail at birth and during adulthood (Fig 4E–4G). In addition, investigation of muscle revealed the presence of eGFP^+^ muscle spindles (Fig 4H), suggesting proprioceptive axons in the muscle spindles are still evident with eGFP expression, albeit these neurons mostly expressed dim eGFP in their soma (Fig 2E and 2F). Sections taken from the limbs of newborn pups reveal that both deep thick axon bundles and fine superficial nerve endings are detected by eGFP fluorescence (Fig 4I). In the hind limb footpad, both autonomic axons innervating the sweat glands and the sensory nerve endings in the epidermis are eGFP^+^ (Fig 4J and 4K). Fine details of skin innervation were evident even after 2 years of age (i.e. P800; Fig 4K inset), and the axons of sensory neurons were visualized with high precision and clarity, without a need for immunocytochemical labeling.

Our findings suggest that UCHL1-eGFP reporter line would be most appropriate to visualize and study peripheral innervation of the skin, and that it would be informative about the changes in intra-epidermal nerve fiber density. To test this hypothesis, we performed two different experiments within the context of diabetes and ALS.

### Visualization of epidermal innervation density in a diabetes model

**Fig 5:**
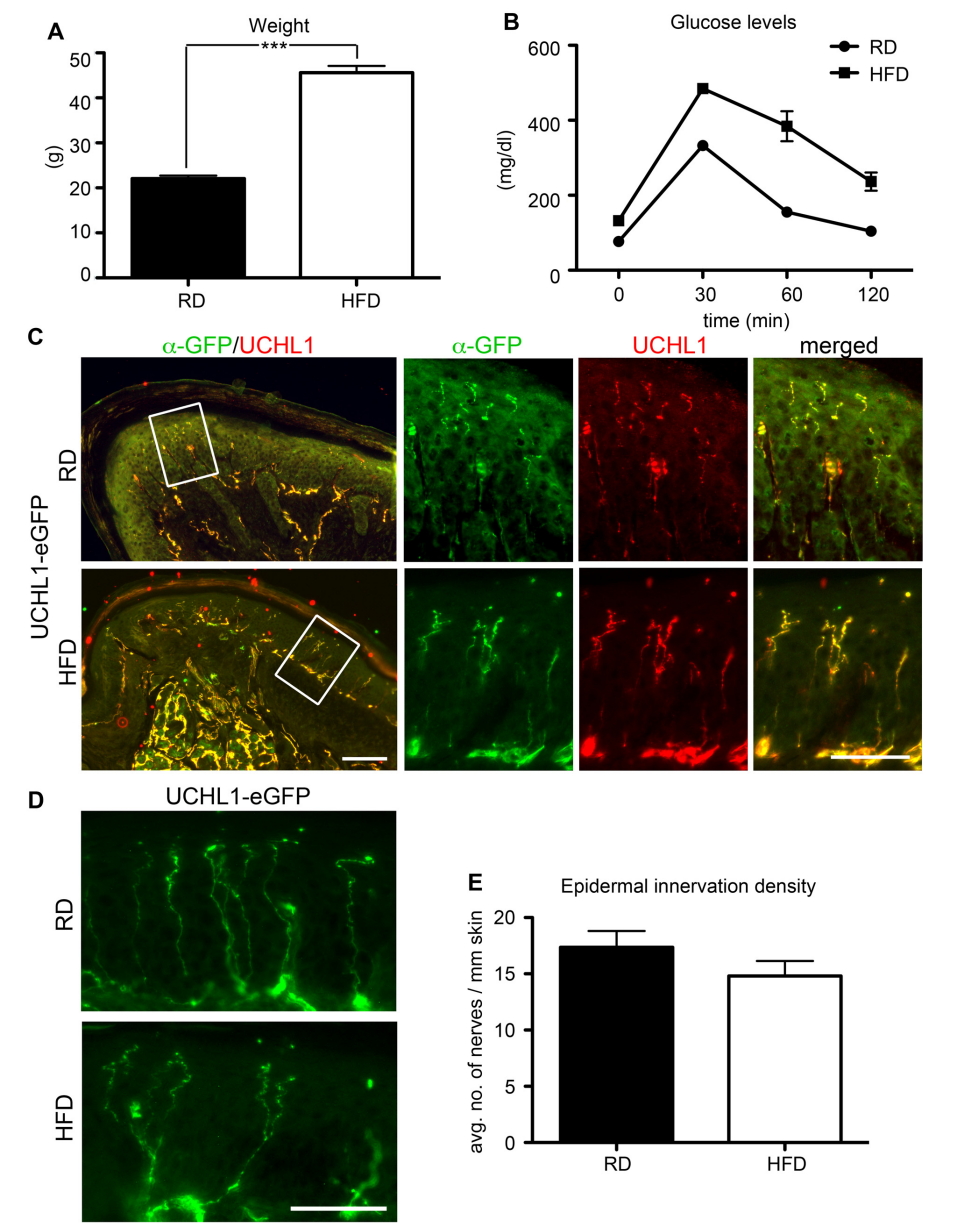
UCHL1-eGFP reporter mice reveal epidermal innervation density in diabetic mice. (A) Average weight of UCHL1-eGFP reporter mice on regular diet (RD) and high fat diet (HFD) for 5 months. (B) Glucose tolerance test showing blood glucose levels of UCHL1-eGFP mice on RD and HFD at 0, 30, 60 and 120 min after glucose administration. (C) eGFP expression shows complete co-localization with UCHL1 in the hindlimb footpad in both control and diabetic mice. (D) eGFP^+^ axons reveal no significant change in epidermal innervation density at 5 months in early-diabetic mice. (E) Bar graph representation of average number of eGFP^+^ nerves per mm length of footpad epidermis in UCHL1-eGFP mice on RD and HFD. Bar graphs represent mean ± SEM. Student’s *t*-test, *** *P* < 0.001. Scale bars C 250 μm; C inset, D 50 μm.

UCHL1 immunohistochemistry is extensively used to study peripheral axonopathy in mouse models of diabetes, primarily looking at the footpad innervation [19,29,30]. To determine whether UCHL1-eGFP reporter mice are a suitable tool to investigate peripheral axons in a mouse diabetes model, they were placed on a high fat diet (HFD) at 6 weeks to induce diabetes. At 22 weeks, mice on a HFD had significantly higher body weight compared to mice on a regular diet (RD) (Fig 5A; 22 ± 0.7 g in RD, *n* = 4 mice; 45.6 ± 1.5 g in HFD, *n* = 5 mice; *P* < 0.0001). To confirm that diabetes was established in mice placed on HFD, we performed glucose tolerance test (GTT). While UCHL1-eGFP mice on RD were able to return to baseline blood glucose levels 2 hrs after glucose administration, mice on HFD had significantly higher blood glucose levels, a sign of type II diabetes (Fig 5B; blood glucose levels 2h after administration: 104 ± 0.85 mg/dl in RD, *n* = 6 mice; 250 ± 3.28 mg/dl in HFD, *n* = 5 mice; *P* = 0.0009). eGFP^+^ axons were used to assess the extent of footpad skin innervation in the HFD-induced mouse diabetes model. eGFP and UCHL1 expression fully overlapped in the epidermis of the footpad of both control (RD) and diabetic (HFD) mice (Fig 5C). We compared the hind limb footpad epidermal innervation density (average number of eGFP^+^ axons per mm length of epidermis) between HFD and RD mice. In line with previous reports [29,30], we found that after 20 weeks on HFD, epidermis started to show early signs of reduction in innervation density, albeit it did not reach significant levels (Fig 5D and 5E; 17.36 ± 1.44 axons in RD, *n* = 6 mice; 14.78 ± 1.35 axons in HFD, *n* = 5 mice; *P* = 0.23), as expected.

### Epidermal innervation density is reduced during disease end-stage in ALS

**Fig 6:**
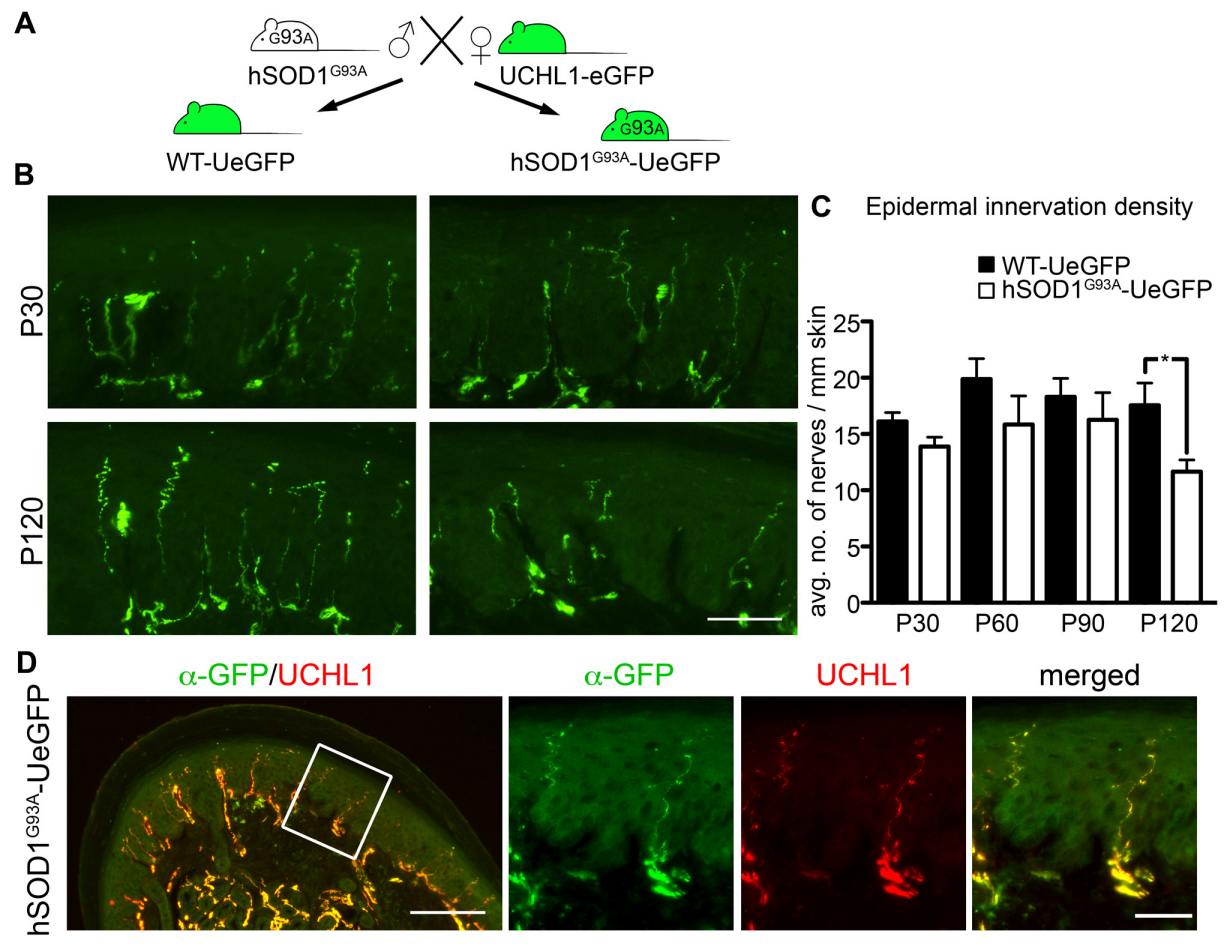
The epidermal innervation density is reduced only during disease end-stage in ALS. (A) UCHL1-eGFP reporter mice were crossed with hSOD1^G93A^ mice to generate the hSOD1^G93A^-UeGFP ALS reporter mice and WT-UeGFP control mice. (B) eGFP^+^ axons are comparable at P30 (presymptomatic stage) between ALS reporter and control animals, but reveal a reduction in epidermal innervation density at P120 (end-stage) only in the hSOD1^G93A^-UeGFP mice. (C) Bar graph representation of average number of eGFP^+^ nerves per mm length of footpad epidermis in WT-UeGFP and hSOD1^G93A^-UeGFP mice at P30, P60, P90 and P120. (D) eGFP shows complete co-localization with UCHL1 expression in the hindlimb footpad of P120 (end-stage) hSOD1^G93A^-UeGFP mice. Bar graphs represent mean ± SEM. Student’s *t*-test, * *P* < 0.05. Scale bars B, D inset 50 μm; D 200 μm.

By crossing UCHL1-eGFP female reporter line to male hSOD1^G93A^ mice, we generated hSOD1^G93A^-UeGFP ALS reporter line [18] (Fig 6A). Peripheral neuropathy has been reported in some patients [31–36] and in ALS models [37,38]. To investigate the potential involvement of the sensory nervous system defects to disease pathology, and to determine the timing and extent of changes with respect to disease progression, we compared the innervation density of hind limb footpad in hSOD1^G93A^-UeGFP and WT-UeGFP mice, at P30 (pre-symptomatic), P60 (early-symptomatic), P90 (late-symptomatic) and P120 (end-stage) [39]. At P30, P60, and P90 the average number of intraepidermal nerve fibers innervating the skin were comparable between WT-UeGFP and hSOD1^G93A^-UeGFP mice (P30: 16% ± 1 axons in WT-UeGFP, *n* = 6 mice, 14 ± 1 axons in hSOD1^G93A^-UeGFP, *n* = 7 mice; P60: 21 ± 3 axons in WT-UeGFP, *n* = 3 mice, 16 ± 3 axons in hSOD1^G93A^-UeGFP, *n* = 3 mice; P90: 18 ± 2 axons in WT-UeGFP, *n* = 3 mice, 16 ± 2 axons in hSOD1^G93A^-UeGFP, *n* = 3 mice). However, by P120, skin innervation was significantly impaired only in the hSOD1^G93A^-UeGFP mice (Fig 6B and 6C; 18 ± 2 axons in WT-UeGFP, *n* = 6 mice; 12 ± 1 axons hSOD1^G93A^-UeGFP, *n* = 6 mice; *P* = 0.0254). The reduction in epidermal innervation density cannot be explained by reduced eGFP expression, because eGFP and UCHL1 are both present in the distal axons innervating the footpad (Fig 6D).

Our report demonstrates that eGFP expression under the UCHL1 promoter genetically labels sensory neurons with a genetic fluorescent tag that is stable and long-lasting. The UCHL1-eGFP reporter line can be used as a tool to visualize peripheral neurons, their axon outgrowth and target innervation patterns during development, adulthood and within the context of disease. Being able to visualize sensory and motor neurons in the same mouse is especially important, and our studies with the diabetes model and with hSOD1^G93A^-UeGFP mice demonstrate the versatile use of UCHL1-eGFP reporter line in two different disease paradigms.

## Discussion

UCHL1 is emerging as one of the most interesting proteins in the nervous system. UCHL1 is a unique deubiquitinating enzyme with both hydrolase and ligase activities, and is required for maintaining ubiquitin levels in neurons [40]. It can bind and stabilize monoubiquitin, hydrolyze polyubiquitin, and add ubiquitin to already ubiquitinylated proteins [5,40,41]. Even though UCHL1 expression is particularly high in the PNS, it displays a more restricted expression profile in the CNS, and is predominantly present in CSMN [18].

Due to its high-level expression in the sensory nervous system [8,9], sympathetic nervous system [7,9], ENS [10–12], retina [9,13], as well as neuroendocrine cells [7], UCHL1 expression has been extensively used as a marker to label and study peripheral neurons within the context of development, disease, injury and repair. But this required isolation of tissue and performing immunocytochemistry. Therefore, various reporter mouse lines were generated to visualize and study the PNS. Transgenic mice expressing yellow fluorescent protein (YFP) under the control of the *Thy-1* promoter label various neuron types, including different subsets of cortical neurons, spinal motor neurons, sensory and sympathetic neurons, and retina [42]. Thy1-YFP mice allowed *in vivo* imaging of cutaneous innervation [43] and regenerating nerve fibers [44–46]. In addition, axonopathy was observed in a multiple sclerosis disease model [47]. Using the human peripherin locus to drive the expression of eGFP, a Peripherin-eGFP reporter line was generated, which labeled sensory neurons during development and in the adult animal, as well as spinal motor neurons, enteric neurons and retinal ganglion cells [48]. A similar expression pattern was observed in the Peripherin-eGFP reporter line generated using the mouse locus [49].

Due to high-level expression and long-term presence of UCHL1 in the sensory neurons, generating a reporter line using the *UCHL1* promoter became more desirable. UCHL1-Histone2BmCherry:GFP-gpi double reporter mice were generated using a BAC containing the UCHL1 locus. In this reporter line Histone2B-mCherry labels the nuclei of UCHL1 expressing cells red, and GFP-gpi labels the cell membrane, including axonal projections, green [50]. As expected, sensory and sympathetic chain ganglia and enteric neurons were labeled in this reporter line, allowing sorting neuronal progenitors in fetal intestines using flow cytometry [50]. A UCHL1-LacZ knock-in mouse line (UCHL1^tm1Dgen^) is also available that acts as a reporter, but has UCHL1 haploinsufficiency, since one copy of the gene is replaced with *LacZ* [51].

Each of these reporter lines has their strength and weaknesses based on the experimental paradigm and the neurons of interest. Here we show that UCHL1-eGFP reporter line recapitulates many aspects of the endogenous UCHL1 expression pattern during development and adulthood, and it offers important advantages: 1) eGFP expression is present in DRG, TG, nodose, sympathetic and parasympathetic ganglia, enabling visualization of their target innervation, and circuitry formation; 2) eGFP gene expression starts early and persists beyond two years in the mouse, allowing visualization and detailed investigation of neuron populations both during early embryonic development and at later stages in life; and 3) Since eGFP is also expressed in motor neurons, this reporter line for the first time allows investigations of sensory and motor defects in the same animal, *in vivo*. Since eGFP expression recapitulates that of UCHL1 *in vivo*, and can be detected without the need of processing samples for immunohistochemistry, the UCHL1-eGFP reporter line offers many advantages. For example, it could be especially useful for diseases that affect the PNS, such as neuropathic pain, trigeminal neuralgia and sensory ataxia, as well as axon injury/regrowth and wound healing studies.

UCHL1 immunohistochemistry in combination with cutaneous nerve and skin biopsy has proven invaluable for clinical diagnosis of peripheral neuropathy [52–54]. Of particular interest, peripheral innervation of the epidermis is known to be impaired with progression of diabetes. UCHL1 expression is used to mark sensory neurons and their axons to study axonopathy in biopsy samples from patients, revealing a correlation between disease and extent of axonal degeneration [55–57]. UCHL1 is highly conserved between mice and humans, and thus its expression in mouse models is used to model and understand disease paradigms in patients [58–60]. For example, mice injected with streptozotocin (SZT) developed diabetes, and UCHL1 expression revealed significant axonopathy by 6 months [61]. More related to our study, the HFD induced diabetes model revealed no difference at 14 [30] and 16 weeks [29], but significant reductions were observed by 34 weeks [62]. We selected 20 weeks of HFD to investigate if indeed significant denervation occurs between 16 and 34 weeks, and found early signs of denervation; supporting previous findings that peripheral axonopathy is a progressive condition in diabetes.

Being able to label both sensory and motor neurons in the same mouse is very important. Motor neuron circuitry, which degenerates in many diseases, receives significant input from the periphery. Therefore, to understand the involvement of the PNS defects in ALS and other motor neuron diseases, it is important to reveal the timing and extent of PNS defects in line with motor dysfunction and circuitry degeneration. UCHL1-eGFP mice offer great advantages for such studies. Crossing UCHL1-eGFP with hSOD1^G93A^ mice, we generated the hSOD1^G93A^-UeGFP transgenic mouse model of ALS, and showed that this disease reporter recapitulates previously reported CSMN degeneration in the hSOD1^G93A^ mice [18,39]. In this study, we investigated cutaneous innervation, and quantified the epidermal innervation density. Our findings confirm findings in some ALS patients, and reveal the occurrence of axonopathy in the ALS mouse, albeit at later stages. This finding is in support of recent findings suggesting a potential involvement of sensory nervous system degeneration in ALS [32,36]. Sensory axon loss and reduction in epidermal nerve density in skin biopsies from ALS patients has been reported [31–34,63–65]. Moreover, the severity of axonal pathology correlated with disease duration [33] and site of onset [64]. One study reported a 46% reduction in the number of large diameter sensory neurons in L5 DRGs of ALS patients [35]. In the dorsal roots of end-stage hSOD1^G93A^ mice, 30% of the sensory axons were degenerating, without a decrease in DRG numbers [37,38]. These studies suggest vulnerability of peripheral neurons in motor neuron diseases, but the extent and timing of such potential degeneration awaits further studies.

We recently generated the UCHL1-eGFP reporter mice [18]. CSMN in the motor cortex and the degeneration resistant SMN in the spinal cord are genetically labeled with eGFP expression. Here we characterize the eGFP expression in the PNS of this reporter line, and show that all sensory neurons are genetically labeled with eGFP expression starting from embryonic development to late adulthood. More importantly, crossing this reporter line to disease models generates reporter lines of diseases in which both the motor and the sensory neurons are genetically labeled. Our study suggests that UCHL1-eGFP mice it could be especially useful to study diseases that affect the PNS, and hSOD1^G93A^-UeGFP mice would be ideal to study the cellular and molecular aspects of PNS dysfunction in line with motor neuron defects.

## Supporting Information

S1 ARRIVE Checklist ARRIVE Checklist. (PDF)
